# Morphological and functional regionalization of trunk vertebrae as an adaptation for arboreal locomotion in chameleons

**DOI:** 10.1098/rsos.221509

**Published:** 2023-03-29

**Authors:** Julia Molnar, Akinobu Watanabe

**Affiliations:** ^1^ Department of Anatomy, New York Institute of Technology, College of Osteopathic Medicine, Old Westbury, NY 11568, USA; ^2^ Division of Paleontology, American Museum of Natural History, New York, NY 10024, USA; ^3^ Life Sciences Department, Natural History Museum, London, SW7 5BD UK

**Keywords:** chamaeleonidae, vertebrae, biomechanics, arboreality, modularity

## Abstract

Regionalization of the vertebral column can help animals adapt to different kinds of locomotion, including arboreal locomotion. Although functional axial regionalization has been described in both chameleons and arboreal mammals, no morphological basis for this functional regionalization in chameleons has been proposed. However, recent studies have described regionalization in the presacral vertebral column of other extant squamates. To investigate possible morphological regionalization in the vertebral column of chameleons, we took morphometric measurements from the presacral vertebrae of 28 chameleon species representing all extant chameleon genera, both fully arboreal and ground-dwelling, and performed comparative analyses. Our results support chameleons exhibiting three or four presacral morphological regions that correspond closely to those in other sauropsids, but we detected evolutionary shifts in vertebral traits occurring in only arboreal chameleons. Specifically, the anterior dorsal region in arboreal chameleons has more vertically oriented zygapophyseal joints, predicting decreased mediolateral flexibility. This shift is functionally significant because stiffening of the anterior thoracic vertebral column has been proposed to help bridge gaps between supports in primates. Thus, specialization of existing morphological regions in the vertebral column of chameleons may have played an important role in the evolution of extreme arboreal locomotion, paralleling the adaptations of arboreal primates.

## 1. Introduction

Arboreal locomotion presents common challenges and has produced many convergent morphological and kinematic adaptations in animals that inhabit trees. For example, arboreal primates and some marsupials exhibit increased forelimb length and range of motion in protraction, scapular mobility, grasping appendages and modification of the vertebral column (e.g. [1]). Morphological specializations of the pre-sacral vertebral column have been linked to arboreal locomotion and even specific behaviours including bridging, cantilevering and leaping. A wide range of arboreal mammals, including primates, show stiffening of the axial skeleton, thought to assist in bridging gaps between supports [2]. In particular, the thoracolumbar region in mammals that habitually use cantilevering and bridging in an arboreal setting exhibits reduced intervertebral spacing, thought to increase sagittal stability [3]. Similarly, lumbar vertebrae in arboreal marsupials show morphological specializations such as dorsoventrally expanded vertebral bodies and curved articular facets, thought to resist sagittal bending while allowing lateral flexion and rotation [4]. Tree shrews, in contrast, show increased dorsoventral flexibility in the thoracolumbar region, which may increase their versatility in placing their feet on irregular substrates [5]. Among strepsirrhine primates, species that primarily use slow arboreal quadrupedalism and/or suspension have lumbar vertebrae that are morphologically distinct from those of leaping strepsirrhines [6]. However, because most of our understanding of arboreal locomotion is based on studies of primates and other small-bodied mammals, it is unclear to what extent non-mammalian tetrapods share these adaptations.

Chameleons present a good opportunity to study the relationship between axial regionalization and arboreality. Like primates, arboreal chameleons exhibit adaptations for locomotion on narrow, discontinuous substrates such as grasping appendages, decreased lateral undulation, increased forward reach, mobile pectoral girdles and forearm pronation [7–9]. In addition, there is reason to believe that axial regionalization may be present in some chameleons as part of their adaptation for arboreal locomotion. Although reptiles lack the extreme heterogeneity and abrupt transitions that characterize the mammalian vertebral column (e.g. [10–12]), at least three pre-sacral morphological regions can be detected in most extant amniotes [13]. Whereas the presacral vertebral column of tegus contains five morphological regions but only two that are biomechanically distinct [11], at least two functionally distinct trunk regions have been detected in veiled chameleons (*Chamaeleo calyptratus*) [8]. Finally, extant chameleons show a range of arboreal behaviours from extreme arboreal chameleons that spend their lives almost entirely in trees to mainly ground-dwelling chameleons that inhabit the forest floor and low bushes [14,15]. Therefore, comparative vertebral morphology of chameleons could reveal whether the functional regions observed in veiled chameleons coincide with morphological regions and how precaudal regionalization (if present) relates to locomotor behaviour or arboreality.

To explore the relationship between vertebral morphology, regionalization, phylogeny and arboreal locomotion in chameleons, we took morphometric measurements from presacral vertebrae. Previous studies using mechanical joint testing, correlation with locomotor behaviour, and engineering beam theory have shown relationships between vertebral morphometrics and function in vertebrates including crocodiles, marine mammals and primates [6,12,16–19]. Pre-zygapophyseal angle, defined as the angle between cranial articular facets in cranial view, is one of the best predictors of intervertebral joint mobility. Larger pre-zygapophyseal angles (indicating more horizontally oriented facets) are associated with decreased stiffness and increased range of motion in lateral flexion, and pre-zygapophyseal angles greater than 90° are thought to indicate a vertebral column specialized for mediolateral bending [6,12,16]. Also associated with increased stiffness and/or decreased range of motion in the mediolateral direction are mediolaterally broader vertebral bodies and transverse processes [6,12,17]. In turn, greater stiffness and smaller ranges of motion in dorsoventral bending are associated with dorsoventrally taller vertebral bodies and neural spines. Craniocaudally longer vertebral bodies and narrower laminae are associated with an overall increase in compliance and range of motion [6,18,19]. Thus, variation in these metrics between species and across the vertebral column can indicate biomechanical specialization for different behaviours and environments.

Based on these relationships between morphometrics and function, we predicted that the arboreal and ground-dwelling chameleons show particular morphometric patterns related to their locomotor behaviour. In arboreal chameleons, we predicted that the anterior trunk vertebrae would show morphometric characteristics associated with decreased mediolateral compliance and range of motion, such as more vertically oriented articular facets and mediolaterally broader vertebral bodies. The rationale is that restricted lateral undulation in the anterior dorsal region has been reported in several arboreal chameleon species [8,20]. By contrast, we predicted that the ground-dwelling chameleons would show a more uniform morphology along the trunk and vertebral measurements associated with low compliance and small ranges of motion in both dorsoventral and mediolateral directions, such as craniocaudally shorter vertebral bodies and broader laminae. Our reasoning was that, qualitatively, ground-dwelling chameleons exhibit very little body undulation during locomotion, and behaviourally they have not been observed performing acrobatic manoeuvres like their arboreal relatives. Finally, we predicted that the emergence of a specialized anterior dorsal region most likely coincided with the radiation of large-bodied arboreal chameleons, which occurred during the Eocene epoch [21]. Because arboreal chameleons are thought to form a clade that emerged relatively late in chameleon evolution and diversified rapidly [21], morphological innovations related to a fully arboreal mode of life most likely appeared early in the history of this lineage.

## 2. Methods

### 2.1. Specimens

Micro-computed tomography (CT) scans of 28 chameleons (19 arboreal chameleons and nine ground-dwelling chameleons) representing all extant chameleon genera were downloaded from Morphosource (www.Morphosource.org) (table 1). A scan of the common agama (*Agama agama*) was included in the analysis as an outgroup because it is a member of the family Agamidae, the sister group to Chamaeleonidae. Scan data with high resolution and good contrast were selected.

**Table 1.** Specimens and scans sampled in this study. All files were downloaded from www.Morphosource.org. The second column indicates ancestral habitat structure according to Tolley *et al*. [21], Higham *et al*. [22] and da Silva and Tolley [23]. ‘Thick perch’ refers to forests and woodlands and ‘fine perch’ refers to shrubs, grasslands and heaths. Institutional abbreviations: California Academy of Sciences (CAS), Field Museum of Natural History (FMNH), Harvard University Museum of Comparative Zoology (MCZ), United States National Museum of Natural History (USNM), University of Texas at El Paso (UTEP), Alexander Koenig Research Museum (ZFMK).

species	habitat	specimen	media ID	resolution (mm)
*Agama agama* (outgroup)	ground-dwelling	FMNH: Amphibians and reptiles: 188910	ark:/87602/m4/M53004	0.0879
*Brookesia perarmata*	ground-dwelling	ZFMK 62848	ark:/87602/m4/M39276	0.0351
*Brookesia superciliaris*	ground-dwelling	USNM: Amphibians and reptiles: 163509	ark:/87602/m4/M53244	0.0260
*Brookesia thieli*	ground-dwelling	CAS: Herp: 126309	ark:/87602/m4/M59737	0.0262
*Palleon nasus*	ground-dwelling	MCZ: Herp: r-189661	ark:/87602/m4/M52095	0.0350
*Rhampholeon platyceps*	ground-dwelling	USNM: Amphibians and reptiles: 160320	ark:/87602/m4/M50852	0.0260
*Rhampholeon spectrum*	ground-dwelling	USNM: Amphibians and reptiles: 570676	ark:/87602/m4/M50851	0.0260
*Rieppeleon brevicaudatus*	ground-dwelling	UF: Herp: 65355	ark:/87602/m4/M11272	0.0308
*Rieppeleon kerstenii*	mixed^a^	CAS: Herp: 151163	ark:/87602/m4/M71909	0.0296
*Archaius tigris*	arboreal (thick perch)	MCZ: Herp: r-3080	ark:/87602/m4/M52049	0.0346
*Bradypodion melanocephalum*	arboreal (fine perch)	CAS: Herp: 156358	ark:/87602/m4/M50710	0.0278
*Bradypodion pumilum*	arboreal (thick perch^b^)	CAS: Herp: 167761	ark:/87602/m4/M30919	0.0239
*Bradypodion thamnobates*	arboreal (thick perch)	CAS: Herp: 156719	ark:/87602/m4/M50714	0.0278
*Calumma amber*	arboreal (thick perch)	USNM: Amphibians and reptiles: 149828	ark:/87602/m4/M50848	0.0346
*Calumma brevicorne*	arboreal (thick perch)	USNM: Amphibians and reptiles: 163511	ark:/87602/m4/M50835	0.0350
*Calumma parsonii*	arboreal (thick perch)	CAS: Herp: 126310	ark:/87602/m4/M61865	0.0355
*Chamaeleo calyptratus*	arboreal (thick perch)	CAS: Herp: 135515	ark:/87602/m4/M50726	0.0260
*Chamaeleo gracilis*	arboreal (thick perch)	MCZ: Herp r-96837	ark:/87602/m4/M52069	0.0350
*Chamaeleo zeylanicus*	arboreal (thick perch)	CAS: Herp: 232061	ark:/87602/m4/M33037	0.0346
*Furcifer lateralis*	arboreal (thick perch)	MCZ: Herp: r-11593	ark:/87602/m4/M52583	0.0346
*Furcifer pardalis*	arboreal (thick perch)	USNM: Amphibians and reptiles: 157349	ark:/87602/m4/M53246	0.0260
*Furcifer verrucosus*	arboreal (thick perch)	USNM: Amphibians and reptiles: 149341	ark:/87602/m4/M52747	0.0260
*Kinyongia carpenteri*	arboreal (thick perch)	UTEP: Herp: 20370	ark:/87602/m4/M50744	0.0282
*Kinyongia tavetana*	arboreal (thick perch)	CAS: Herp: 103653	ark:/87602/m4/M30924	0.0239
*Kinyongia xenorhina*	arboreal (thick perch)	MCZ: Herp: r-47254	ark:/87602/m4/M52009	0.0346
*Nadzikambia mlanjensis*	arboreal (thick perch)	USNM: Amphibians and reptiles: 145580	ark:/87602/m4/M50858	0.0260
*Trioceros feae*	arboreal (thick perch)	CAS: Herp: 207667	ark:/87602/m4/M33041	0.0346
*Trioceros jacksonii*	arboreal (fine perch)	CAS: Herp: 66019	ark:/87602/m4/M63957	0.0355
*Trioceros quadricornis*	arboreal (fine perch)	CAS: Herp: 104554	ark:/87602/m4/M50840	0.0260

^a^This species from mixed habitats was grouped with ground-dwelling taxa for the purposes of this study.

^b^*B. pumilim* contains a woodland morph that uses thick perches and a heathland morph that uses fine perches [22]. This specimen was collected from Stellenbosch, South Africa (https://www.idigbio.org/portal/records/74cb192f-2a27-4d47-8a00-c71a40a04f40), which is a woodland locality [22], so it is classified as thick perch habitat.

### 2.2. Segmentation and measurements

**Figure 1.**
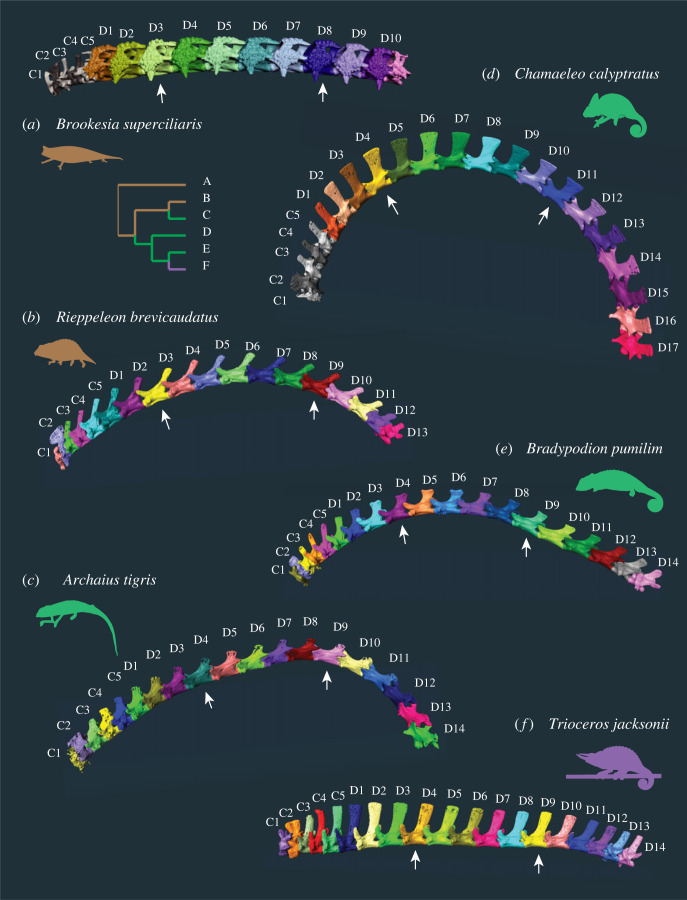
Vertebral morphology in representative species illustrating variation within the family Chamaeleonidae. Segmented cervical and dorsal vertebrae in left lateral view from two ground-dwelling chameleons (*a,b*) and four arboreal chameleons (*c–f*). Both the number and shape of vertebrae vary among taxa; for example, the Jackson's chameleon *T. jacksonii* has 17 dorsal vertebrae with elongated neural spines, whereas the bearded leaf chameleon *B. superciliaris* has only 10 dorsal vertebrae characterized by very short neural spines and expanded, heavily ossified transverse processes. Cladogram shows relationships between taxa and ancestral habitat reconstruction (brown = ground-dwelling; green = arboreal thick perch; purple = arboreal fine perch) based on Tolley *et al*. [21]. Silhouettes represent taxa. C, cervical; D, dorsal.

**Figure 2.**
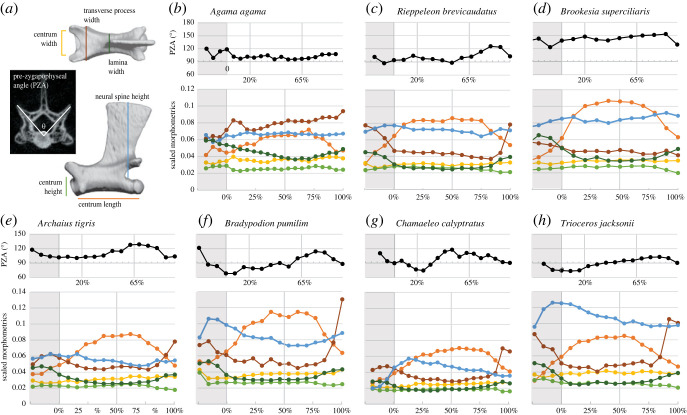
Vertebral measurements in representative study taxa. Measurements taken from vertebrae (*a*) and patterns of change throughout the cervical and dorsal vertebral column (*b–h*). (*b–h*) show angular (top) and scaled linear (bottom) morphometrics by taxon: outgroup (*b*), ground-dwelling chameleons (*c,d*), and arboreal chameleons (*e–h*), including the secondarily arboreal *A. tigris*. The three groups have characteristically different patterns, particularly in pre-zygapophyseal angle (black) and neural spine height (blue). Horizontal axes correspond to location along the dorsal vertebral column, where 0% represents the cervical–dorsal transition, 100% represents the dorsal–sacral transition and 50% indicates the division between anterior and posterior dorsal regions. Locations of representative anterior and posterior trunk vertebrae (20% and 65%) are marked. Grey shading indicates the cervical region; the proatlas and axis (C1–2) have been excluded.

Reconstructed scans were imported into Amira v. 2020.2 (www.thermofisherscientific.com). Morphometric measurements were taken digitally from meshes based on segmented micro-CT scans. Taking measurements digitally from micro-CT scans allowed us to take precise measurements from very small structures and to reconstruct slices through the joints and measure the angles of articular facets. First, the entire skeleton was globally segmented using the threshold tool (figure 1), and the numbers of cervical and dorsal vertebrae were counted. Cervical vertebrae were defined as those between the skull and the first vertebra whose ribs contacted the sternum [24]. No *a priori* distinction was made between thoracic and lumbar vertebrae because chameleons lack a rib-free ‘lumbar’ region. Seven morphometric measurements were taken in Amira from all postaxial cervical and dorsal vertebrae, focusing on metrics that have been linked to function in previous studies. Linear measurements (centrum length (CL), width (CW) and height (CH); neural spine height (NSH), transverse process width (TPW) and lamina width (LW)) were taken from the mesh surfaces (figure 2*a*) using the three-dimensional measure tool. Pre-zygapophyseal angles were measured using the three-dimensional angle tool from transversely oriented slices taken through the zygapophyseal facets using the Slice tool in Amira.

### 2.3. Statistical analysis

To test whether vertebral morphologies were statistically different by vertebral region or habitat, we compared metrics taken from two representative vertebrae from each taxon. Taxa were coded as ground-dwelling or arboreal (table 1) based on categories used by Tolley *et al*. [21]. One chameleonid (*Rieppeleon kerstenii*) is found in more than one habitat, but we included it among the ground-dwelling taxa because it does not exhibit the extreme arboreal lifestyle of most ‘true’ chameleons [25]. Vertebrae from two regions were compared: an anterior dorsal vertebra approximately 20% along the trunk (excluding the cervical region) and a posterior dorsal vertebra approximately 65% of the way along the trunk (figure 1). These regions were chosen to match regions with greater and lesser amplitudes of dorsolateral undulation during locomotion in a previous study of *Chamaeleo calyptratus* [8]. Because size disparity is observed between ground-dwelling and arboreal taxa, linear measurements were scaled to trunk length to correct for body size prior to statistical analysis: each measurement was divided by the summed centrum lengths of dorsal vertebrae. Total morphometric variance along the vertebral column (excluding C1–2) was calculated for each specimen as a sum of the variance for each linear variable (all in mm), after the variables had been scaled by trunk length to correct for potential allometric signal in disparity. Variance in pre-zygapophyseal angle was also calculated. For statistical comparisons between ground-dwelling and arboreal taxa, we performed generalized least-squares analysis in R [26] on these morphometric and variance data, with phylogenetic correction when appropriate.

To evaluate regionalization patterns within each species, we followed Jones *et al*. [11] to fit segmented regression models on morphometric distance measurements (CL, CW, CH, TPW, NSH, LW) collected from cervical and dorsal vertebrae. The proatlas and axis (C1 and C2) were excluded because they lack vertebral bodies and/or neural spines, so equivalent measurements collected for other presacral vertebrae could not be taken from these vertebrae. Additionally, the primary analysis of regionalization excluded pre-zygapophyseal angles to maintain equivalency in measurement (mm) for all variables considered in the analysis. However, we also conducted a regionalization analysis based on principal coordinates analysis that includes both unscaled and size-scaled linear measurements and pre-zygapophyseal angle (electronic supplementary material, figure S2). We extracted first three principal components (PC) from the morphometric data that account for greater than 95% of the total variation within each specimen. For regionalization analysis based on linear measurements and pre-zygapophyseal angle (PZA), we used the first seven PC axes. In all regionalization analyses, we allowed up to five regions, which is the maximum number that has been reported for tetrapods [10,27,28]. Through this analysis, we generated the most supported number of vertebral regions based on Akaike information criterion (AIC), as well as diagrams illustrating the regions along the vertebral column.

### 2.4. Comparative methods

For phylogenetically informed analyses, we used a time-calibrated phylogeny based on Tolley *et al*. [21]. To detect significant evolutionary shifts in neural spine height (NSH) and PZA in representative anterior dorsal and posterior dorsal vertebrae, we used the ‘runSurface’ function [29] in the ‘surface’ R package [30] to fit Ornstein–Uhlenbeck (OU) model of trait evolution and detect significant shifts in stabilizing trait regime. Neural spine height was scaled to summed centrum lengths of dorsal vertebrae to account for body size differences. We then identified the best supported model based on stepwise AIC comparisons.

## 3. Results

**Figure 3.**
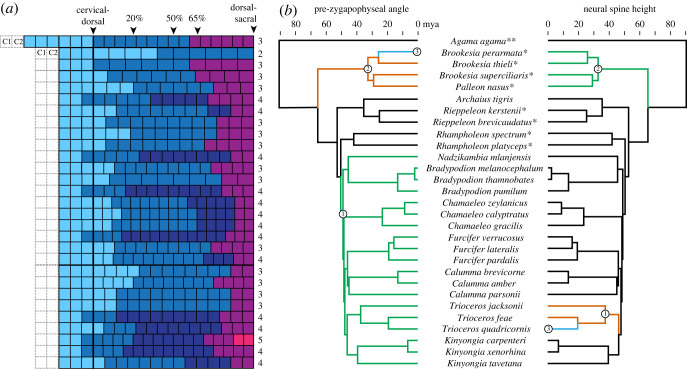
Inferred axial regionalization and evolutionary shifts in pre-zygapophyseal angle and neural spine height across the chameleon family tree. (*a*) Diagram depicting the regionalization pattern along the vertebral series for each species, showing the greatest support for 3–4 morphological regions in chameleons. The column of numbers to the right shows the best supported number of regions based on AIC of segmented regression fitting. Proatlas and axis (C1–C2) were not included in the analysis. (*b*) Evolutionary shifts in pre-zygapophyseal angle (left) and neural spine height (right) in representative anterior and posterior dorsal vertebrae (located approximately 20% and 65% along the length of the dorsal column, respectively) mapped on time-calibrated phylogeny of species sampled for this study. Asterisks denote outgroup squamates (**) and ground-dwelling chameleons (*) according to [17]. The results indicate a shift in PZA close to the origin of most extant arboreal chameleons (node 2) and additional shifts in the node leading to the earliest-diverging ground dwelling clade *Brookesia* + *Palleon* (node 1) and within that clade (node 3). Shifts in NSH evolution occurred around the origins of the genera *Brookesia* (node 2) and *Trioceros* (node 1), and within *Trioceros* (node 3). Circled numbers denote nodes at which shifts occur, and each branch colour represents different optimal trait values for each metric based on fitted OU model.

Compared with their ground-dwelling relatives, the vertebral column of arboreal chameleons has a greater number of presacral vertebrae and displays more variation between anterior and posterior regions. The ground-dwelling chameleons tend to have fewer presacral vertebrae than the arboreal chameleons, but there is some overlap in vertebral count (15–19 versus 18–23, respectively) (figure 3*a*). All else being equal, fewer vertebrae would produce a less flexible vertebral column. For nearly all chameleons we sampled, a three- or four-region model including cervical, anterior dorsal and posterior dorsal regions was most strongly supported. No phylogenetic or functional pattern in regionalization was obvious (figure 3*a*). The most obvious morphometric differences among species are pre-zygapophyseal angle (PZA) and neural spine height (NSH). Arboreal chameleons show a peak in NSH at the cervical–dorsal transition, but no major differences among groups were evident in the rest of the presacral vertebral column (electronic supplementary material, figure S1). However, the anterior dorsal region in arboreal chameleons has significantly more dorsoventrally oriented zygapophyses (electronic supplementary material, table S2), suggesting greater mediolateral stiffness and increased leverage for dorsal extension. The evolutionary shift toward specialization of the anterior dorsal vertebrae most likely occurred around the ancestral node of arboreal chameleons (figure 3*b*).

### 3.1. Morphological modularity

Two to five morphological regions were optimally supported across sampled species, and no phylogenetic pattern was obvious. Three regions were detected in *Agama,* roughly corresponding to the cervical (light blue), anterior dorsal (dark blue) and posterior dorsal (purple) regions found in other extant sauropsids [27] (figure 3*a*). Most chameleons (26 of 28 species) had either three or four regions. Of the 12 chameleons with four regions, five species had a two-vertebra ‘anterior cervical’ region, which may reflect the difference between C3, which does not bear ribs, and C4–C5, which do. Of the remaining seven species with four regions, six had a two-vertebra ‘lumbar’ region, and one had a three-vertebra ‘cervical–dorsal transition’ region. The chameleon with five regions (*Kinyongia carpenteri)* had both anterior cervical and lumbar regions. One chameleon, *Brookesia perarmata,* had only two regions with a division near the midpoint of the dorsal region. *Brookesia perarmata* also has the fewest presacral vertebrae [15] (figure 3*a*). Inclusion of PZA in the regionalization model through principal coordinates analysis resulted in more variation in both the number of regions and the location of divisions between regions (electronic supplementary material, figure S2). However, no clear phylogenetic or functional pattern emerged.

### 3.2. Morphological variation along the presacral vertebral column

Although vertebral regionalization in squamates is less distinct than it is in mammals, the anterior and posterior dorsal regions in arboreal chameleons differ in several ways from those of ground-dwelling chameleons and other limbed squamates. Contrary to previous descriptions [31], the zygapophyseal facets of trunk vertebrae in chameleons generally are oriented more mediolaterally than dorsoventrally, except in the anterior dorsal regions of many arboreal chameleons, where more dorsoventrally oriented facets are common (electronic supplementary material, figure S1). NSH at the cervical–dorsal transition was greater in the arboreal chameleons than the ground-dwelling chameleons, whereas in both groups NSH decreased slightly from the anterior dorsal to posterior dorsal regions. Qualitatively, the size, shape and inclination of neural spines vary across taxa: for example, *Chamaeleo* and *Bradypodion* possess neural spines that are sub-rectangular and dorsoventral in orientation, whereas *Trioceros* shows dorsally elongated neural spines (figure 1*d–f*). Ground-dwelling chameleons show greater variation in neural spine morphology, ranging from the narrow, posteriorly inclined spines of *Rieppeleon* to the roughened accessory bridges of bone in *Brookesia* (figure 1*a,b*)*.* No difference in the relative dimensions of the vertebral bodies or laminae between ground-dwelling and arboreal chameleons was apparent. Differences in vertebral morphometrics between regions and taxa was investigated further using phylogenetically corrected ANOVA (reported below) and models of trait evolution for NSH and PZA (see Evolutionary models section). Centrum length was not modelled because it closely parallels number of vertebrae.

As in most squamates [31], zygapophyseal joints in *Agama* and the ground-dwelling chameleons are oriented more mediolaterally than dorsoventrally, reflecting specialization for mediolateral mobility. Pre-zygapophyseal angle in *Agama* lacks clear regional trend and remains close to 100° throughout the trunk (figure 2*b*). In most of the ground-dwelling chameleons, the zygapophyses are even more horizontally oriented. By contrast, the PZA in arboreal chameleons in the anterior dorsal regions is generally less than 90°. When we directly compared PZA of representative anterior and posterior dorsal vertebrae (located approx. 20% and 65% along the length of the dorsal column, respectively), differences in PZA between regions were statistically significant (*p*-value < 0.05), while the *p*-value associated with differences between arboreal and ground-dwelling groups is slightly above the 0.05 threshold for statistical significance. Mean PZA in the anterior dorsal vertebrae of arboreal chameleons (mean (*M*) = 85.1°, s.d. = 10.4°) was smaller than in the posterior dorsal vertebrae of arboreal chameleons (*M* = 100.2°, s.d. = 10.1°; *p* < 0.001). In ground-dwelling chameleons the difference in angles between anterior and posterior dorsal vertebra was not statistically significant: PZA of anterior dorsal vertebrae (*M* = 118.8°, s.d. = 24.2°) was not significantly different from PZA of posterior dorsal vertebrae (*M* = 119.1°, s.d. = 17.1°; *p* = 0.875). After accounting for the effect of phylogenetic relatedness, PZA in the anterior dorsal region of arboreal chameleons was smaller than that of ground-dwelling chameleons but fell just short of statistical significance at the 0.05 level (*p* = 0.056). No other metric demonstrated statistically notable differences between arboreal and ground-dwelling chameleons (0.12 < *p* < 0.97; electronic supplementary material, table S1).

In addition to more vertically oriented pre-zygapophyses, the arboreal chameleons share a pattern of increasing neural spine height (NSH) around the cervical–dorsal transition followed by a decrease throughout the dorsal region (figure 2*e–h*; electronic supplementary material, figure S1). *Archaius*, thought to have independently acquired an arboreal lifestyle [21], is an exception, showing little difference in NSH between the cervical and dorsal regions. In *Trioceros* species*,* neural spines are tall relative to centrum length throughout the trunk (figure 2*h*; electronic supplementary material, figure S1). The ground-dwelling chameleons and *Agama* lack a sharp peak at the cervical–dorsal transition, and NSH decreases slightly or shows no trend throughout the trunk. *Palleon,* the earliest-diverging genus of chameleons, is the only ground-dwelling taxon in which we observed a pronounced peak at the cervical–dorsal transition (electronic supplementary material, figure S1). In both ground-dwelling and arboreal chameleons, size-corrected NSH was slightly greater in the anterior dorsal vertebrae than the posterior dorsal ones, but the differences were not statistically significant (0.22 < *p* < 0.412; electronic supplementary material, table S2). For ground-dwelling chameleons, the mean NSH was 2.1 mm (s.d. = 0.7 mm) in the anterior region versus 1.8 mm (s.d. = 0.7 mm) in the posterior region, and for arboreal chameleons mean NSH was 4.8 mm (s.d. = 2.5 mm) in the anterior region versus 4.0 mm (s.d. = 2.1 mm) in the posterior region. NSH (scaled to trunk length) was not significantly different between arboreal and ground-dwelling taxa at either the anterior or posterior position after accounting for phylogenetic relatedness (0.61 < *p* < 0.68).

Common to chameleonids and *Agama* is an increase in centrum length toward a peak in the mid-dorsal region followed by a decrease toward the posterior dorsal region (figure 2*b–h*; electronic supplementary material, figure S1). In arboreal chameleons, centra were significantly longer in the posterior dorsal region than the anterior dorsal region (*p* = 0.011; electronic supplementary material, table S2). Proportionally longer centra are associated with regions of increased flexibility [18], suggesting that differences in both centrum length and pre-zygapophyseal angle in the posterior dorsal region of arboreal chameleons contribute to a regional increase in flexibility. However, centrum length relative to trunk length was not significantly different between arboreal and ground-dwelling chameleons after correcting for phylogenetic relatedness (0.377 < *p* < 0.484; electronic supplementary material, table S1), and, because summed centrum length was used to correct for body size, this metric was closely tied to number of dorsal vertebrae. Cervical vertebrae in all the squamates we examined are distinguished from dorsal vertebrae by anteroposteriorly shorter centra and broader laminae, although the cervicodorsal transition is less pronounced in *Agama* than in the chameleons.

All the squamates we studied share a peak in transverse process width (TPW) in the cervical region and a sharp increase in the posterior dorsal region (figure 2*b–h*; electronic supplementary material, figure S1). In the dorsal region in *Agama,* the transverse processes are relatively broad (TPW exceeds centrum length), and they increase in lateral breadth throughout the trunk. In the chameleons, however, the transverse processes remain relatively narrow throughout the dorsal region.

Finally, disparity along the presacral vertebral column (excluding the proatlas and axis) was not significantly different between arboreal chameleons and ground-dwelling chameleons (0.24 < *p* < 0.68; electronic supplementary material, table S3). However, arboreal chameleons have a greater number of dorsal vertebrae on average; the same amount of disparity between individual vertebrae could produce larger differences between regions.

### 3.3. Evolutionary models

Based on best supported models of stabilizing trait evolution, evolutionary shifts in PZA most likely occurred at the node leading to arboreal chameleons and in an early diverging lineage of ground-dwelling chameleons. The earliest shift occurred at the node leading to *Brookesia* + *Palleon* (figure 3*b*, node 1), which have larger PZAs (110–153°; electronic supplementary material, table S4), and finally in *B. perarmata*, which has PZAs smaller than most other ground-dwelling taxa but larger than most arboreal ones (110–113°; electronic supplementary material, table S4)*.* The second shift occurred at the node representing the most recent common ancestor of arboreal chameleons, excluding *Archaius* (figure 3*b*, node 2), corresponding to the regional increase in PZA noted in the morphometric analysis. The OU model fitted to NSH scaled to trunk length showed shifts in the ground-dwelling clade including *Brookesia* and *Palleon* and in the node leading to *Trioceros* and within that genus, which includes the only fine-branch arboreal specialists in our analysis. Both shifts tended toward longer neural spines relative to trunk length.

## 4. Discussion

We hypothesized that morphological correlates of decreased mediolateral compliance and range of motion would be found in the anterior trunk vertebrae of arboreal chameleons, and that this distinction most likely emerged at the origin of true arboreal chameleons. Morphometric data from 28 chameleon species representing all extant genera support this prediction, showing that the anterior dorsal region of arboreal chameleons exhibits a characteristic pattern of decreased PZA, correlated with decreased mediolateral mobility. Further, analysis of individual vertebrae from two locations along the vertebral column showed that PZA from the anterior dorsal vertebrae in arboreal chameleons is significantly smaller than posterior dorsal vertebrae in the same group and also smaller than anterior dorsal vertebrae in ground-dwelling chameleons, often resulting in facet joints that are oriented more vertically than horizontally. That pre-zygapophyseal angles smaller than 90° are found only in arboreal chameleons and almost exclusively in the anterior part of the presacral vertebral column suggests that more vertical facet joints play a role in climbing, possibly supporting the pectoral girdle and forelimb during bridging or stabilizing it against mediolateral excursions. When we mapped PZA onto a chameleon phylogeny, an evolutionary shift in facet orientation was recovered at the node leading to a radiation of large-bodied arboreal chameleons. Arboreal chameleons also showed increased neural spine height around the cervical–dorsal transition, possibly reflecting differences in locomotion and/or feeding. Analysis of morphological regionalization showed no clear change in the number and distribution of regions. As such, the trait evolution in arboreal chameleons seems to result from increased morphological differences between existing regions rather than an increase in regionalization.

### 4.1. Chameleon locomotor system

The family Chamaeleonidae includes the most arboreal of all known limbed squamates. Arboreal chameleons regularly navigate substrates narrower than their own bodies and perform acrobatic manoeuvres that require large ranges of motion in both forelimb protraction and trunk flexion, such as reversing direction on a single perch and crossing large open space between perches [20]. Despite their ability to perform tight turns, chameleons employ relatively little lateral undulation of the vertebral column during normal locomotion. While lateral undulation contributes about 10% to body progression in chameleons [8] it contributes 33–52% in terrestrial limbed squamates [32]. Fischer *et al*.'s [8] study of *C. calyptratus* moving on a perch found that this lower contribution to step progression results mainly from restriction of undulation to the posterior region of the spine. They measured lateral flexion between five segments of the dorsal vertebral column, each corresponding to roughly 20% of trunk length (fig. 2 in [8]) and found that angles between the posterior two segments were more than twice as large as between the anterior two segments. Fischer *et al*. [8] demonstrated that functional regionalization of the vertebral column characterizes the locomotion of at least some chameleons, but the current study is the first to propose a morphological basis for this biomechanical difference.

### 4.2. A morphological basis for evolution of extreme arborealism in chameleons

Although chameleons all share morphological specializations for arboreal locomotion such as prehensile tails and digits, their transition to fully arboreal way of life appears to be a relatively recent innovation. The family Chamaeleonidae originated in the Late Cretaceous period (90 Ma), whereas the origin of the clade including most fully arboreal species is thought to have occurred during the Eocene epoch (47 Ma) [21] (figure 3*b*). Ancestral reconstruction indicates that the earliest chameleons most likely were ground-dwelling and that arboreal habits were independently acquired in the Eocene radiation and in *Archaius* [21]. Therefore, evolutionary shift in PZA in the clade including most fully arboreal chameleons may be related to the expansion of forest environments due to increased temperatures during the Early Eocene (*ca* 51–53 Ma) [21], as an adaptation for an extreme arboreal way of life that allowed these chameleons to take full advantage of the arboreal niche.

The evolutionary shift we observed in PZA is especially suggestive of a change in function, because zygapophyseal orientation is considered to be one of the best osteological clues to axial flexibility and mechanics, as close contact between zygapophyses constrains the range and direction of movement of the intervertebral joint [19,33]. More vertically oriented zygapophyses (PZA < 90°) restrict lateral movements of the spine, whereas more horizontally oriented facets (PZA > 90°) restrict sagittal movements [17]. In fact, the mammalian vertebral column is often divided based on zygapophyseal orientation into a pre-diaphragmatic region with more horizontally oriented zygapophyses and a post-diaphragmatic region with vertically oriented zygapophyses, and sagittal bending is thought to be restricted to the latter region [17,34] (but see [35,36] for caveats). In support of this conceptual framework, several studies on extant animals have demonstrated a relationship between zygapophyseal orientation and axial function or behaviour. Among primates, lorises have relatively horizontally oriented facets in the lumbar region, related to an emphasis on lateral movements that facilitate bridging, climbing and reaching behaviours [6]. In crocodylians, more horizontal zygapophyses are found in regions of the spine that predominantly experience lateral movements [16] and are correlated with decreased intervertebral joint stiffness in lateral flexion [12]. Because different axial structures limit movement in different animals [37], caution should be used when generalizing relationships between morphology and function broadly across clades.

While the proposed link between PZA and arboreal locomotion is relatively straightforward, patterns of variation in NSH could be explained by more than one evolutionary hypotheses. The regional increase in NSH observed at the cervical–thoracic junction in most arboreal taxa may increase the leverage of muscles that attach to the pectoral girdle (e.g. trapezius, levator scapulae), facilitating the girdle rotation that contributes substantially to step length in arboreal chameleons [20,38]. In addition, the first four or five dorsal vertebrae form the origin for the latissimus dorsi, a powerful muscle that contributes to limb retraction during climbing [20,38], and taller neural spines would increase the length and possibly the working range of this muscle, contributing to the large anterior–posterior range of motion of the shoulder noted in arboreal chameleons [8,38] (kinematics of ground-dwelling chameleons have not been reported in the literature). Greater NSH also would increase the leverage of muscles that extend the neck and head [6], possibly helping the animal to position its head for ballistic feeding. Taller neural spines might be especially advantageous for feeding in large-bodied chameleons (all of which are arboreal) to accommodate a heavier head. Considering only the representative anterior and posterior dorsal vertebrae sampled in this study, shifts toward longer neural spines were detected in both ground-dwelling and arboreal clades (*Brookesia* and *Trioceros,* respectively). The shift in *Brookesia* + *Palleon* may be related to the elaboration of the vertebral processes in some *Brookesia* species: in *B. superciliaris* and *B. thieli,* the anterior and posterior articular processes are connected by a bridge of bone, and there is an accessory arch between the left and right anterior articular processes that is connected to the spinous process [39]. The functional consequences of this configuration are unknown. In *Trioceros,* the extreme elongation the neural spines in the trunk could be related to locomotion on very narrow substrates (found in open habitats like grassland, heath and small bushes as opposed to forest) as several *Trioceros* species including *T. jacksonii* and *T. quadricornis* are considered fine-branch specialists [21]. However, although *Bradypodion* also includes fine-branch specialists (e.g. *B. melanocephalum*)—probably representing recent radiation into open habitats [40,41]—no evolutionary shift in neural spine height was detected in this genus. Future locomotor studies of these groups would be useful toward exploring relationships between morphology and function across the diversity of extant chameleons.

Chameleons and primates have in parallel acquired morphological and behavioural adaptations that allow them to navigate substrates narrower than their body width including decreased axial flexion. For animals that use slow arboreal locomotion, possible benefits to restricting undulation in the anterior dorsal region include minimizing head movement while stalking prey [8], decreasing the tendency to topple sideways from narrow perches [20] and bridging large gaps in the substrate [2]. Primates with reduced axial flexibility often possess vertebral features such as reduced intervertebral spacing, short vertebral bodies, short lumbar regions and dorsoventrally oriented spinous processes [3,42], whereas we did not observe these specializations in chameleons, although centrum length was slightly but significantly shorter in the anterior region of arboreal taxa (intervertebral spacing and neural spine inclination were not measured). Modification of PZA in chameleons may perform a similar functional role as these morphological mechanisms do in arboreal mammals.

### 4.3. Functional differentiation through specialization of existing modules

The trait evolution in arboreal chameleons reflects larger morphological differences between existing regions rather than an increase in regionalization. This result supports the hypothesis that morphological regions represent potential for functional differentiation that is not always realized. That is, a presacral vertebral column with multiple modules may have more than one functionally specialized region, but not necessarily so; for example, as mentioned in the introduction, the tegu lizard has four presacral morphological regions but only two functional regions [11]. The anterior and posterior dorsal regions in *Agama* and ground-dwelling chameleons may not be functionally different, while the same two regions in arboreal chameleons may differ widely in terms of range of motion, stiffness, muscle leverage and other functional parameters. The difference between PZA in anterior and posterior dorsal regions as part of an evolutionary shift in PZA in the Eocene radiation of arboreal chameleons may indicate a functional shift in the vertebral column related to their adoption of a fully arboreal way of life.

In summary, a marked evolutionary shift toward reorientation of the zygapophyseal joints of the anterior trunk region in the lineage leading to extant large-bodied fully arboreal chameleons seems to have stiffened the anterior part of the trunk against mediolateral movements. This finding parallels a similar shift in vertebral morphology and function thought to help arboreal primates bridge large gaps between branches. Combined with previous kinematic data showing restricted lateral undulation in the anterior trunk, these results support the hypothesis that specialization of existing morphological regions was part of the suite of adaptations that allowed extant ‘true’ chameleons to adopt an extreme arboreal lifestyle.

## Data Availability

Raw measurements are provided in supplementary tables S4 [43]. Scans from which measurements were taken are freely available on Morphosource.com (Table 1 for media IDs). The time-calibrated phylogeny (a subset of the tree from Tolley *et al*. [21]) is provided as a Nexus file in electronic supplementary material.
